# Number of Sprint Repetitions in a Sprint Interval Training Session Does Not Moderate Improvements in V̇O_2_max With Training: A Systematic Review and Meta‐Analysis

**DOI:** 10.1111/sms.70347

**Published:** 2026-07-20

**Authors:** M. Hutchinson, D. J. Kinghorn, E. C. R. Hall, R. S. Metcalfe, D. R. Paval, I. J. Gallagher, N. B. J. Vollaard

**Affiliations:** ^1^ Faculty of Health Sciences and Sport University of Stirling Stirling UK; ^2^ Applied Sports, Technology, Exercise and Medicine Research Centre (A‐STEM), Swansea University Swansea UK; ^3^ Centre for Biomedicine & Global Health, School of Applied Sciences, Edinburgh Napier University Edinburgh UK

**Keywords:** cardiorespiratory fitness, exercise prescription, meta‐analysis, sprint interval training, V̇O_2_max

## Abstract

Sprint interval training (SIT) is promoted as a time‐efficient method for improving maximal aerobic capacity (V̇O_2_max); however, the “classic” protocol (4–6 × 30‐s all‐out sprints) is less time‐efficient than claimed, may be poorly tolerated, and is expected to lead to low adherence. The present study examined the effect of the number of sprint repetitions per session on training‐induced changes in V̇O_2_max through meta‐analysis of the available literature. PubMed and Web of Science were searched (May 2016—September 2024) for eligible studies involving ≥ 2 weeks of training involving repeated all‐out cycling with pre‐ and post‐training V̇O_2_max data. Forty‐four eligible studies were combined with 34 studies from a previous meta‐analysis, totalling 93 trials across 78 studies (*n* = 1188). A hierarchical random‐effects meta‐regression model was used to assess the influence of key moderators on the change in V̇O_2_max. SIT produced an 8.3% increase in V̇O_2_max (95% CrI: 7.0 to 9.5%). Increasing sprint repetitions showed a trivial association with changes in V̇O_2_max (−0.04% per additional sprint, 95% CrI: −0.32 to 0.24%). Increasing intervention duration was associated with the largest improvements in V̇O_2_max (0.7% per additional week; 95% CrI: 0.3 to 1.0%). The present study confirms that increasing sprint repetitions does not meaningfully enhance V̇O_2_max improvements in untrained populations. As performing fewer sprint repetitions increases time‐efficiency and attenuates reductions in affect, future research into interventions for improving general health in physically inactive populations should focus on low‐volume SIT protocols.

**Trial Registration:** PROSPERO database (registration number CRD42022349104)

## 1 Introduction

A large proportion of individuals worldwide do not meet current physical activity (PA) recommendations based on moderate‐ or vigorous‐intensity continuous exercise (MICT) [1, 2]. While lack of time is frequently cited as a barrier to participation [1, 2, 3], it is also recognized that behavioral, environmental, and motivational factors play an important role in determining exercise engagement [4]. As such, identifying exercise strategies that are both effective and feasible remains an important goal for improving population health. This has led to an increased focus on identifying effective but time‐efficient alternative exercise interventions for improving health, such as (sub‐)maximal high‐intensity interval training (HIIT) and supramaximal sprint interval training (SIT). In a number of meta‐analyses, HIIT and SIT have been shown to be effective at improving the key health marker of maximal aerobic capacity (V̇O_2_max). The importance of V̇O_2_max for health is emphasized by multiple studies [5, 6, 7, 8, 9] showing that every 1‐metabolic equivalent (MET; ~3.5 mL kg^−1^ min^−1^) increase in V̇O_2_max is associated with a reduced risk ranging from 12%–35% of all‐cause mortality in men and women. Improvements in V̇O_2_max following SIT are often comparative to those achieved with MICT, along with other important health markers such as blood pressure, blood lipids, and insulin sensitivity, although not consistently greater across all studies [10, 11, 12, 13, 14, 15]. Despite its efficacy, the practical relevance of SIT has been widely disputed. Much of this criticism has been directed at “classic” SIT involving protocols involving 4–6 × 30‐s all‐out sprints, which may be perceived as physically demanding and unpleasant [16, 17]. In addition, most SIT studies have been conducted using cycle ergometers capable of delivering supramaximal resistance, which may limit the generalisability of findings to settings without access to specialized equipment [18]. Furthermore, although SIT is frequently promoted as a time‐efficient exercise strategy, the extent to which reducing training time alone is sufficient to improve adherence remains unclear.

These considerations have led to increased interest in identifying lower‐volume SIT protocols that retain physiological benefit while potentially improving feasibility [18]. For example, we and others have shown that the reduced‐exertion high‐intensity interval training (REHIT) protocols involving as few as two 20‐s all‐out sprints [19, 20, 21, 22, 23, 24, 25, 26] or three 20‐s all‐out sprints [27, 28, 29, 30, 31, 32] within a 10‐min low‐intensity cycling session. This raises an important question regarding the dose–response relationship of SIT: does increasing the number of sprint repetitions within a session enhance the adaptive response, or is there a minimum effective dose beyond which additional work provides little benefit?

No studies investigating the effects of the classic SIT protocol have provided a specific justification for the use of 4–6 × 30‐s all‐out sprints [33]. A previous meta‐analysis from our group reported that reducing the number of sprint repetitions did not attenuate improvements in V̇O_2_max [34]. This finding is important, as reducing the number of sprint repetitions has a large effect on total training time commitment, making the exercise more time‐efficient [18]. Reducing the number of sprints also substantially attenuates the reduction in affective valence during exercise, making the exercise less unpleasant and more acceptable [35]. However, the previous meta‐analysis was limited by the relatively small number of studies employing low‐volume protocols. Since then, the number of available studies has increased substantially, providing an opportunity to re‐examine the question. In addition, the previous meta‐analysis employed Magnitude‐Based Inference, an approach that has subsequently been the subject of considerable methodological debate regarding the interpretation of uncertainty and statistical evidence [36, 37, 38, 39] Therefore, the objective of the present meta‐analysis was to determine whether increasing the number of sprint repetitions within a SIT exercise protocol enhances the improvements in V̇O_2_max in untrained individuals, using a robust statistical analysis approach.

## 2 Materials and Methods

### 2.1 Literature Search and Screening Process

This study has been registered on the PROSPERO database (registration number CRD42022349104) and was carried out in accordance with the guidelines of Preferred Reporting Items for Systematic Reviews and Meta‐Analyses [40]. Online databases Web of Science and PubMed were searched for relevant journal articles with a date range from 01/05/2016 to 30/09/2024—articles from prior to 01/05/2016 were taken from the previous meta‐analysis [34]. The search strategy combined independent variable terms such as “Wingate”, “all‐out”, “sprint” and “interval training” with dependent variable terms including “fitness”, “aerobic capacity”, “aerobic power”, “V̇O_2_max”, “VO_2_peak”, “oxygen uptake” and “oxygen consumption”. These were combined using Boolean logic (“AND”, “OR”), resulting in a total of 28 search term combinations. The full search strategy, including all Boolean combinations and search terms, is provided verbatim in File S1 to ensure reproducibility. Study selection was guided by PICOS criteria relating to population (untrained adults), intervention (repeated all‐out cycling sprints), comparator (pre‐post design or control group), outcome (V̇O_2_max), and study design (experimental studies). Studies were excluded if: (1) exercise was not all‐out, sprints were < 10 s, or intervention duration was < 2 weeks, (2) participants involved clinical populations or athletes, (3) mean participant age was < 18 years or mean baseline V̇O_2_max was > 55 mL kg^−1^ min^−1^, (4) the exercise intervention involved any exercise other than cycling or used SIT in conjunction with another intervention, (5) publication type was a review, letter, or commentary, or (6) the manuscript was not written in English. Studies involving < 10 s sprints were excluded to ensure consistency in the training stimulus, as shorter sprint protocols (e.g., repeated sprint training or Tabata‐style exercise) are characterized by different physiological demands and work: rest structures compared to traditional SIT.

**FIGURE 1:**
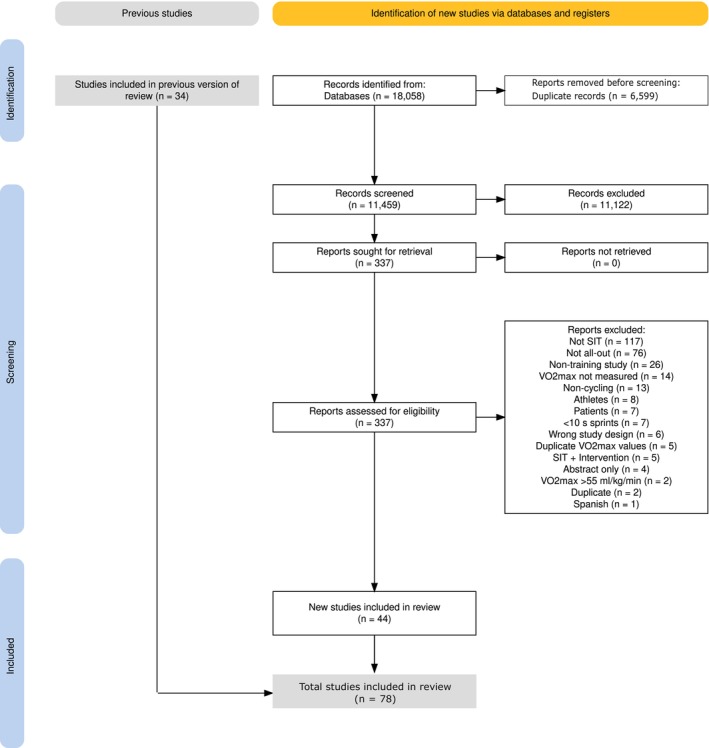
PRISMA flow diagram of the study selection process.

After the removal of 6599 duplicates, the titles and abstracts of 11 459 journal articles were independently screened for eligibility using systematic review software Rayyan [41] by two authors (MH, NBJV) who were blinded during this part of the screening process. Full‐text versions of the 337 remaining potentially eligible articles were reviewed for inclusion into the meta‐analysis (Figure 1). The authors were then unblinded and any discrepancies during the screening process were resolved through discussion. If the dispute(s) could not be resolved, a third reviewer (RM) was used to determine the final decision. The final data set of 44 studies selected for inclusion from the literature search in this study was combined with the studies compiled from a previous meta‐analysis on the same topic [34]. The previous meta‐analysis contained 34 studies from all years up to May 1, 2016, using an identical search strategy. This resulted in a combined data set of 78 studies for the present systematic review and meta‐analysis.

### 2.2 Data Extraction

To enable the integration of studies identified in the previous meta‐analysis [34], with newly identified studies, the data extraction procedures used in the present review were largely consistent with those applied previously. All included articles were assessed for mean absolute pre‐ and post‐training V̇O_2_max (L min^−1^) as opposed to relative V̇O_2_max (mL kg^−1^ min^−1^). Absolute V̇O_2_max values were prioritized where available; however, both absolute and relative values were included, as proportional changes were analyzed, allowing comparability across units. As 24 of the 44 new included studies were missing mean absolute V̇O_2_max data, the authors were contacted to request this data. Nine of the 24 authors contacted were able to provide the requested missing data [22, 23, 32, 42, 43, 44, 45, 46, 47]. Relative V̇O_2_max was used for 13 studies where absolute V̇O_2_max could not be obtained [25, 26, 48, 49, 50, 51, 52, 53, 54, 55, 56, 57, 58]. Plot digitizer software (PlotDigitizer; Version 3.1.6) was used to obtain relative V̇O_2_max [14] and absolute V̇O_2_max [31] from the two remaining published journal articles where the relevant values were not stated and could not be obtained. Participant characteristics (sex, age, BMI, and baseline V̇O_2_max), exercise intervention variables (minimum and maximum number of sprint repetitions per exercise session, sprint duration, sprint resistance, sprint/recovery ratio, intervention duration, training frequency per week, and total number of sessions), and study design (controlled, uncontrolled) were extracted from each study as potential moderators. For calculation of proportional changes, if the spread of the data was reported using standard error of the mean (SEM), this was converted to standard deviation (SD) by multiplying the SEM by the square root of the sample size [59].

## 3 Statistical Analysis

### 3.1 Effect Size Calculation

Training responses were quantified as log‐transformed proportional changes in V̇O_2_max:

$$\mathrm{yi}=\log\left(\frac{\text{Post}}{\mathrm{Pre}}\right)$$

Sampling variances were calculated using pre‐ and post‐intervention SD and an imputed pre‐post correlation of *r* = 0.90, based on pre‐post correlation scores typically observed in similar exercise intervention studies carried out in our laboratory. Sensitivity analyses were conducted using *r* = 0.95 and *r* = 0.85 to confirm that results were not meaningfully influenced by this assumption.

### 3.2 Statistical Model

The Bayesian analysis reporting guidelines [60] were followed to provide transparency and reproducibility of the steps carried out throughout the present study. The overall effect of sprint interval training on changes in V̇O_2_max was assessed using a Bayesian hierarchical random‐effects meta‐regression model, which was fit using the *brms* (v2.22.0) [61] package within the statistical computing software R (v.4.3.2, R Core Team [62]). Random intercepts for study and effect nested within‐study were included to account for non‐independence of multiple effects within studies. Weakly informative priors were used as recommended by the developers of the Stan probabilistic language upon which *brms* is based [63], and model diagnostics confirmed appropriate convergence and fit (Figure S1). Full details of model structure, model priors, and sensitivity analyses are reported in File S1. All R scripts and data required to reproduce the analysis are available at: https://github.com/matthutch95/SIT_VO2max_Reproducibility.git.

Study parameters and participant characteristics were added as moderators to the model to assess the influence they each had on the overall pooled effect size. The following moderators were included: number of sprint repetitions, sprint duration (s), intervention duration (weeks), work: rest ratio, baseline relative V̇O_2_max (mL kg^−1^ min^−1^), age (y), weekly training frequency, sex (% male), BMI (kg m^−2^), and study design (controlled vs. non‐controlled). All continuous moderators were mean‐centred prior to analysis to allow interpretation of the intercept as the expected effect at average moderator values. Total weekly sprint time was calculated as sprint repetitions × sprint duration × weekly session frequency, which was run in a separate model to avoid issues with multicollinearity. BMI was examined in a secondary sensitivity analysis due to missing data. All model estimates are reported as posterior means with 95% credible intervals (CrIs) where the true effect is estimated to lie with 95% probability [64]. Conditional effects plots were used to visualize the relationships between effect size and the moderators.

### 3.3 Risk of Bias

A funnel plot was created to visually assess the risk of publication bias. Egger's test was conducted to assess the asymmetry observed in the funnel plot. To further evaluate the potential impact of small‐study effects, the trim‐and‐fill method was applied using a random effects model to estimate the number of potentially missing studies and to obtain an adjusted pooled effect size. These analyses were conducted using the *metafor* [65] package in R. Within‐study risk of bias was evaluated using the Cochrane Risk of Bias V.2.0 tool [66]. To reduce subjective bias and increase the accuracy of the process, two reviewers (MH, NBJV) evaluated the studies independently, with discrepancies discussed after the completion of the assessment. If the discrepancies could not be resolved through discussion, a third reviewer (RM) was used to provide a final judgment on the rating. Risk of Bias was evaluated across 5 domains: the randomization process, deviations from the intended interventions, missing outcome data, measurement of the outcome, and selection of the reported result. The RoB V.2.0 for crossover trials tool [67] was used for any studies that implemented a crossover study design. This tool contains one additional domain: risk of bias arising from period and carryover effects. The Risk of Bias in Non‐randomized studies of Interventions, Version 2 (ROBINS‐I V2, 2024; 7 domains) [68] was used for any studies where participants were not randomized to their intervention.

### 3.4 Sensitivity Analyses

Given the relatively large number of included studies, the posterior estimates were expected to be largely driven by the observed data rather than the prior distributions. To confirm this, a prior sensitivity analysis was performed using wider priors, and the resulting posterior estimates were compared [69, 70] (File S1). A sensitivity analysis was carried out by removing 4 outlier datasets that contained > 10 sprint repetitions, as all these datasets involved short (10–15 s) sprints.

## 4 Results

**FIGURE 2:**
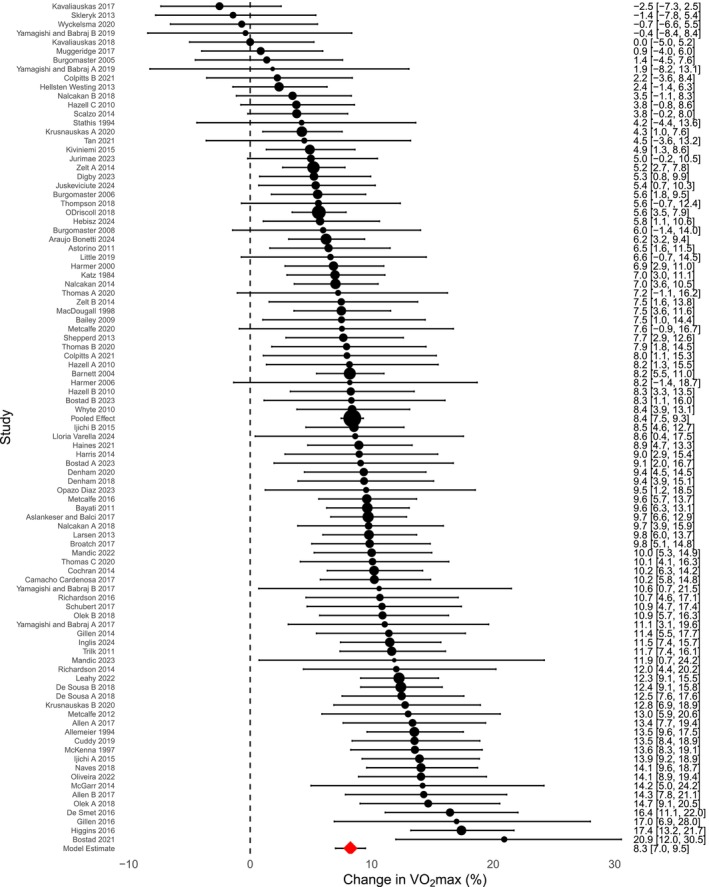
Estimated change in V̇O_2_max following SIT across included studies. Letters A, B, and C used in study labels denote different datasets from within the same study.

**FIGURE 3:**
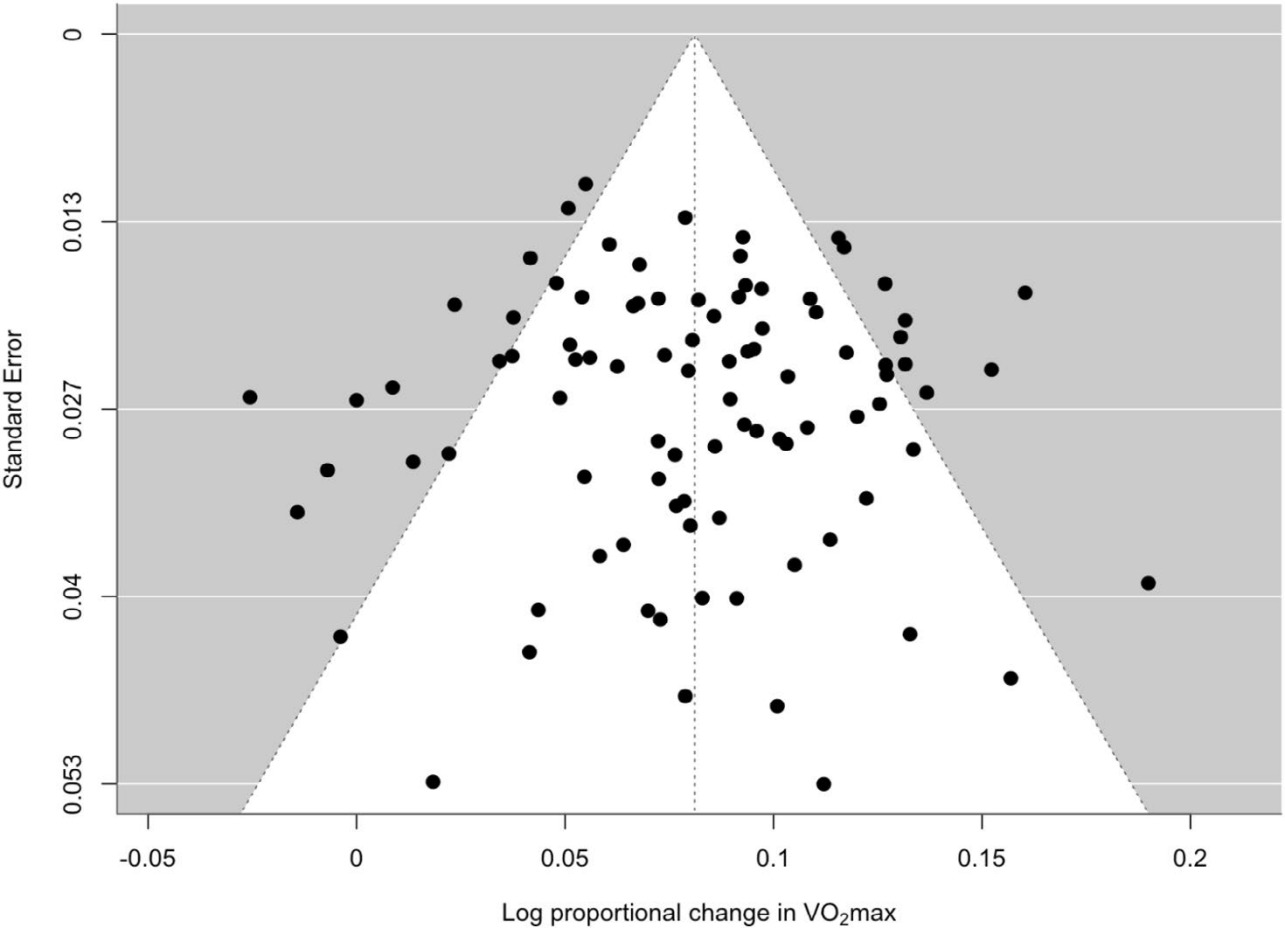
Funnel plot of 93 study effect sizes (black circles). The vertical black dashed line represents the estimated pooled estimate, and the diagonal dashed black lines represent 95% credible intervals.

Data from 78 studies (93 trials; *n* = 1188) are shown in Table 1. On average, SIT was associated with an 8.3% increase in V̇O_2_max (95% CrIs: 7.0 to 9.5%; Figure 2). Between‐study heterogeneity was small (τ_*between*_ = 0.018; 95% CrI = 0.002–0.031; Figure S2b). Within‐study variability (τ_within_ = 0.011; 95% CrI = 0.0005–0.027) was also small. Visual inspection of the funnel plot (Figure 3) did not suggest clear asymmetry. This was supported by Egger's regression test, which showed no evidence of small‐study effects (z = −0.24, *p* = 0.81). The trim‐and‐fill analysis estimated that no studies were missing, and the pooled effect size remained unchanged following adjustment (8.3%, 95% CrI: 7.0% to 9.5%). Together, these findings suggest no evidence that small‐study effects influenced the results of the present meta‐analysis. Model estimates are presented in Table S1. Summary statistics for included moderators are presented in Table 2. Estimated changes in V̇O_2_max across meaningful moderator changes are presented in Table 3. File S1 contains tables and figures from model diagnostic checks and sensitivity analyses. Posterior estimates were virtually identical when using wider priors, indicating that the results were entirely data‐driven and not meaningfully influenced by prior specification (Table S2). Table S3 shows the results of the sensitivity analysis, which excluded 4 outlier studies for sprint repetitions atypical to SIT interventions; overall interpretations remain unchanged. Sensitivity analyses using alternative imputed pre‐post correlation coefficients (*r* = 0.85 and *r* = 0.95) also produced near‐identical estimates, indicating that the results were not meaningfully influenced by the assumed correlation. Equivalent frequentist models produced near‐identical estimates (data not shown), and the R scripts and data required to reproduce the analysis are available at: https://github.com/matthutch95/SIT_VO2max_Reproducibility.git.

**TABLE 1:** Study training intervention protocol parameters, participant characteristics, and training effects.

References	Study Design	N (M/F)	Baseline V̇O_2_max (mL kg^−1^ min^−1^)	Age (yr)	BMI (kg m^−2^)	Training duration (wk)	Total training sessions	Sprint duration (s)	Recovery duration (s)	Resistance (% of BM)	Min sprint reps	Max sprint reps	Change in V̇O_2_max (%)
Metcalfe et al. [20]	C	11 (5/6)	34.2 ± 5.2	25.0 ± 3.0	23.5 ± 4.0	6	18	20	200	7.5	1	2	12.7
Nalcakan et al. [24]	NC	18 (14/4)	34.0 ± 9.0	22.0 ± 4.0	25.3 ± 5.9	6	18	10	220	7.5	2	2	3.5
Aslankeser and Balci [71]	C	8 (0/8)	34.3 ± 0.7	20.4 ± 0.6	—	2	10	30	240	7.5	2	2	9.7
Thomas et al. [23]	NC	14 (8/6)	35.7 ± 4.8	26.0 ± 5.0	24.3 ± 4.2	6	18	20	280	7.5	2	2	7.9
Thomas et al. [23]	NC	12 (9/3)	36.2 ± 4.8	24.0 ± 4.0	26.9 ± 2.4	6	24	20	280	7.5	2	2	7.2
Haines et al. [25]	NC	12 (12/0)	44.7 ± 6.8	21.4 ± 2.4	25.4 ± 1.7	5	15	20	200	7.5	1	2	9.0
Nalcakan et al. [24]	NC	18 (11/7)	39.0 ± 7.0	22.0 ± 2.0	23.8 ± 2.2	6	18	20	200	7.5	2	2	9.8
Metcalfe et al. [19]	C	13 (6/7)	28.0 ± 7.0	46.0 ± 9.0	27.2 ± 4.5	6	18	20	180	5.0	2	2	7.6
Thomas et al. [23]	NC	16 (12/4)	34.4 ± 6.1	26.0 ± 6.0	27.1 ± 4.6	6	12	20	280	7.5	2	2	10.1
Leahy, Dalleck and Ramos [26]	NC	12 (5/7)	25.3 ± 2.9	40.8 ± 10.8	—	8	26	20	180	CAROL	2	2	12.3
Metcalfe et al. [21]	NC	34 (17/17)	35.0 ± 7.4	34.1 ± 9.2	24.6 ± 2.9	6	18	20	200	5	1	2	9.6
Cuddy, Ramos and Dalleck [22]	NC	12 (6/6)	25.3 ± 2.9	40.8 ± 10.8	—	8	26	20	180	—	2	2	13.5
Mandic et al. [72]	NC	9 (4/5)	34.8 ± 6.1	27.0 ± 5.0	—	6	18	30	120	7.5	3	3	12.1
Opazo‐Diaz et al. [29]	NC	9 (9/0)	33.7 ± 5.8	21.1 ± 1.7	23.0 ± 1.5	4	12	20	120	—	3	3	9.5
Bostad et al. [31]	NC	10 (0/10)	33.2 ± 7.2	21.0 ± 4.0	—	12	36	20	120	CAROL	3	3	9.1
Bostad et al. [30]	NC	15 (6/9)	37.1 ± 6.9	21.0 ± 2.0	—	12	36	20	120	7.5	3	3	20.9
Little et al. [32]	NC	16 (9/7)	34.3 ± 7.6	21.0 ± 4.0	24.1 ± 4.9	6	18	20	180	—	3	3	6.6
Gillen et al. [27]	C	9 (9/0)	32.0 ± 7.0	27.0 ± 8.0	27.0 ± 5.0	12	36	20	120	5	3	3	17.3
Mandic et al. [73]	NC	29 (16/13)	41.0 ± 7.8	27.0 ± 5.0	23.5 ± 2.5	6	18	30	120	7.5	3	3	10.0
Ijichi et al. [74]	C	10 (10/0)	47.7 ± 4.8	20.4 ± 0.8	21.0 ± 2.9	4	20	30	600	5	3	3	13.9
O'Driscoll et al. [75]	CC	40 (40/0)	42.5 ± 5.2	21.0 ± 1.7	—	2	6	30	120	7.5	3	3	5.7
Gillen et al. [28]	NC	14 (7/7)	29.5 ± 4.0	30.0 ± 9.0	—	6	18	20	120	5	3	3	11.6
Allemeier et al. [76]	C	11 (11/0)	48.7 ± 6.7	22.7 ± 5.0	—	6	15	30	1200	7.5	3	3	13.5
Bostad et al. [31]	NC	10 (10/0)	41.1 ± 7.3	21.0 ± 3.0	—	12	36	20	120	CAROL	3	3	8.3
Tan et al. [77]	NC	15 (6/9)	38.0 ± 9.3	39.0 ± 13.0	23.3 ± 3.8	6	18	30	120	—	2	4	4.5
de Sousa et al. [51]	C	26 (12/14)	42.9 ± 8.6	23.5 ± 5.1	24.5 ± 3.4	2	6	30	180	7.5	3	4	12.5
de Sousa et al. [51]	C	46 (25/21)	43.4 ± 9.1	23.5 ± 5.1	25.0 ± 4.4	2	6	30	180	7.5	3	4	12.4
Oliveira et al. [56]	NC	21 (0/21)	32.0 ± 7.7	28.8 ± 6.0	25.0 ± 3.2	8	24	30	240	—	4	4	14.1
Colpitts et al. [47]	NC	18 (9/9)	36.4 ± 8.6	41.7 ± 13.3	22.9 ± 1.6	4	12	30	240	7.5	2	4	7.4
Colpitts et al. [47]	NC	16 (6/10)	25.8 ± 5.4	39.3 ± 11.8	34.1 ± 3.8	4	12	30	240	7.5	2	4	2.2
Kavaliauskas, Jakeman and Babraj [50]	C	6 (6/0)	41.5 ± 7.0	32.0 ± 7.0	23.0 ± 2.0	2	6	30	240	7.5	4	4	0.0
Naves et al. [48]	NC	24 (0/24)	32.0 ± 7.2	29.8 ± 6.4	25.2 ± 3.2	8	24	30	240	—	4	4	14.1
Kavaliauskas, Steer and Babraj [49]	CC	8 (0/8)	31.7 ± 3.0	21.0 ± 1.0	23.0 ± 4.5	4	8	30	240	7.0	4	4	−2.5
Harris, Rakobowchuk and Birch [78]	C	6 (0/6)	35.0 ± 3.0	22.0 ± 2.0	23.6 ± 1.8	4	12	30	270	7.5	4	4	9.0
Bayati et al. [79]	C	8 (8/0)	44.6 ± 4.3	25.0 ± 0.8	—	4	12	30	240	7.5	3	5	9.6
Schubert et al. [80]	C	12 (5/7)	32.3 ± 7.1	28.8 ± 7.6	28.4 ± 4.7	4	12	20	120	5.0	3	5	10.9
Thompson et al. [81]	NC	10 (6/4)	43.8 ± 6.3	22.0 ± 3.0	—	4	14	30	240	7.5	4	5	5.6
Kiviniemi et al. [82]	C	13 (13/0)	34.7 ± 3.9	48.0 ± 5.0	25.6 ± 2.7	2	6	30	240	7.5	4	6	4.7
Hazell et al. [83]	C	13 (11/2)	47.0 ± 6.7	24.0 ± 3.2	24.7 ± −	2	6	30	240	10.0	4	6	8.3
Ijichi et al. [74]	C	10 (10/0)	46.8 ± 6.3	21.3 ± 1.6	22.2 ± 1.8	4	10	30	600	5.0	6	6	8.4
Larsen, Befroy and Kent‐Braun [84]	NC	8 (8/0)	25.8 ± 4.0	27.0 ± 3.4	—	2	6	30	240	7.5	4	6	9.8
Yamagishi and Babraj [85]	NC	7 (4/3)	43.9 ± 6.0	23.0 ± 3.0	—	2	6	30	240	7.5	4	6	1.9
Yamagishi and Babraj [85]	NC	7 (5/2)	47.6 ± 7.2	25.0 ± 4.0	—	2	6	30	240	7.5	4	6	−0.4
Shepherd et al. [86]	C	8 (8/0)	41.9 ± 1.8	22.0 ± 1.0	24.8 ± 0.8	6	18	30	270	7.5	4	6	7.6
Olek et al. [45]	NC	7 (7/0)	36.4 ± 1.4	20.1 ± 0.3	23.5 ± 0.5	2	6	10	60	7.5	4	6	14.7
Olek et al. [45]	NC	7 (7/0)	34.8 ± 1.5	20.7 ± 0.2	24.3 ± 0.5	2	6	10	240	7.5	4	6	10.9
Denham et al. [87]	NC	11 (11/0)	43.6 ± 5.9	33.0 ± 9.6	26.8 ± 3.3	6	18	30	240	—	4	6	9.4
Broatch, Petersen and Bishop [88]	NC	8 (8/0)	31.5 ± 7.6	26.0 ± 7.0	25.1 ± 3.7	6	18	30	240	7.5	4	6	9.8
McGarr, Hartley and Cheung [89]	C	8 (6/2)	47.2 ± 11.8	25.0 ± 7.0	—	2	8	30	240	7.5	4	6	14.2
Jurimae et al. [54]	NC	11 (11/0)	36.0 ± 7.1	63.0 ± 8.0	26.1 ± 2.1	3	9	30	240	7.0	6	6	5.0
De Smet et al. [53]	NC	10 (10/0)	53.5 ± 2.6	23.0 ± 3.0	—	5	15	30	270	—	4	6	16.5
Astorino et al. [90]	C	20 (11/9)	43.6 ± −	25.0 ± 4.0	—	2	6	30	300	7.5	4	6	6.3
Muggeridge et al. [43]	C	10 (10/0)	40.6 ± 6.6	26.0 ± 4.0	—	3	9	15	240	7.0	4	6	0.9
Wyckelsma et al. [42]	NC	8 (8/0)	34.5 ± 6.9	64.3 ± 6.0	26.0 ± 2.4	3	9	30	240	—	4	6	−0.7
Whyte, Gill and Cathcart [91]	NC	10 (10/0)	32.8 ± 1.4	32.1 ± 8.7	30.3 ± 3.7	2	6	30	270	6.5	4	6	8.4
Hazell et al. [83]	C	13 (11/2)	47.0 ± 6.7	24.0 ± 3.2	24.7 ± −	2	6	10	240	10.0	4	6	8.5
Burgomaster et al. [92]	C	10 (5/5)	41.0 ± 2.0	23.6 ± 3.2	—	6	18	30	270	7.5	4	6	6.3
Denham et al. [93]	NC	10 (10/0)	42.8 ± 6.2	33.3 ± 10.9	26.8 ± 3.5	6	18	30	240	—	4	6	9.4
Nalcakan [94]	C	8 (8/0)	40.2 ± 4.3	21.7 ± 2.2	25.5 ± 2.3	7	21	30	270	7.5	4	6	7.0
Barnett et al. [95]	C	8 (8/0)	47.6 ± −	20.4 ± 1.2	—	8	24	30	180	—	3	6	8.2
Araujo Bonetti et al. [96]	C	14 (14/0)	35.5 ± 4.1	30.0 ± 5.0	—	5	15	30	240	7.0	3	6	6.3
Inglis et al. [57]	C	14 (7/7)	40.9 ± 6.6	28.0 ± 6.0	—	6	18	30	270	7.0	3	6	11.5
Yamagishi and Babraj [97]	C	9 (7/2)	42.2 ± 5.4	27.0 ± 2.7	—	9	18	15	180	7.5	4	6	11.1
Yamagishi and Babraj [97]	C	8 (5/3)	40.6 ± 9.6	27.5 ± 4.5	—	9	18	30	240	7.5	4	6	10.6
Juskeviciute et al. [58]	NC	10 (10/0)	40.8 ± 6.7	21.6 ± 1.4	24.2 ± 3.1	3	9	30	240	—	4	6	5.4
Digby et al. [55]	NC	9 (6/3)	43.8 ± 6.6	22.0 ± 3.0	25.6 ± 4.9	3	9	30	240	7.5	4	6	5.3
Hazell et al. [83]	C	13 (11/2)	47.0 ± 6.7	24.0 ± 3.2	24.7 ± −	2	6	10	120	10.0	4	6	3.9
Cochran et al. [98]	C	12 (12/0)	50.6 ± 6.0	22.0 ± 2.0	—	6	18	30	240	7.5	4	6	10.3
Zelt et al. [99]	C	12 (12/0)	43.9 ± −	22.0 ± 2.0	26.0 ± 3.0	4	12	15	285	7.5	4	6	7.4
Zelt et al. [99]	C	11 (11/0)	48.6 ± −	23.0 ± 5.0	25.0 ± 3.0	4	12	30	270	7.5	4	6	5.3
Krusnauskas et al. [14]	NC	17 (17/0)	47.0 ± 7.0	25.9 ± 6.4	23.5 ± 2.4	3	9	30	240	7.5	4	6	4.3
Krusnauskas et al. [14]	NC	9 (9/0)	43.9 ± 8.2	25.3 ± 5.4	24.7 ± 3.2	3	9	30	240	7.5	4	6	12.8
Burgomaster, Heigenhauser and Gibala [100]	C	8 (8/0)	48.9 ± 2.1	21.0 ± 1.0	—	2	6	30	240	7.5	4	7	5.6
Bailey et al. [101]	C	8 (5/3)	42.0 ± 6.0	21.0 ± 5.0	—	2	6	30	240	7.5	3	7	7.4
Richardson and Gibson [102]	C	9 (5/4)	40.0 ± −	21.0 ± 1.0	—	2	6	30	240	7.5	4	7	11.2
Higgins et al. [103]	NC	23 (0/23)	29.1 ± 4.8	20.4 ± 1.5	30.3 ± 4.5	6	18	30	240	—	5	7	17.4
Richardson et al. [46]	C	14 (9/5)	42.2 ± 8.6	20.0 ± 1.0	—	2	6	30	240	7.5	4	7	10.7
Burgomaster et al. [104]	C	8 (6/2)	44.6 ± 3.2	22.0 ± 1.0	—	2	6	30	240	7.5	4	7	1.4
Trilk et al. [105]	C	14 (0/14)	21.6 ± 1.1	30.1 ± 6.3	35.7 ± 6.3	4	12	30	240	5.0	4	7	11.7
Katz et al. [106]	NC	8 (8/0)	51.8 ± −	24.2 ± 4.3	—	8	32	30	240	—	8	8	7.0
Scalzo et al. [107]	NC	21 (11/10)	41.5 ± 5.7	22.5 ± 1.0	22.4 ± 0.9	3	9	30	240	7.5	4	8	3.7
Allen et al. [44]	C	20 (7/13)	41.0 ± 4.3	49.2 ± 6.1	27.3 ± 4.0	9	27	30	240	6.5	4	8	14.3
MacDougall et al. [108]	NC	12 (12/0)	50.8 ± 1.8	22.7 ± 2.0	—	7	21	30	180	7.5	4	10	7.5
McKenna et al. [109]	NC	8 (8/0)	47.1 ± 2.6	20.9 ± 0.6	—	7	21	30	180	7.5	4	10	13.7
Stathis et al. [110]	NC	8 (6/2)	49.6 ± 3.6	22.1 ± 1.0	—	7	21	30	180	—	3	10	4.2
Harmer et al. [111]	C	7 (5/2)	43.7 ± 6.2	24.0 ± 5.0	23.8 ± 5.0	7	21	30	180	7.5	4	10	8.2
Harmer et al. [112]	NC	7 (7/0)	49.8 ± 5.9	22.0 ± 3.0	—	7	21	30	180	7.5	4	10	6.9
Camacho‐Cardenosa et al. [52]	C	8 (8/0)	33.1 ± 6.7	24.4 ± 3.5	23.1 ± 2.7	4	8	10	20	—	10	10	10.2
Hebisz et al. [113]	NC	10 (7/3)	47.7 ± 9.2	22.8 ± 2.8	—	5	15	30	90	7.0	6	10	5.8
Skleryk et al. [114]	C	8 (8/0)	29.7 ± 1.3	40.2 ± 2.3	32.2 ± 2.1	2	6	10	80	5.0	8	12	−1.7
Lloria‐Varella et al. [115]	NC	13 (5/8)	37.4 ± 5.6	23.0 ± 5.0	—	6	15	15	120	7.0	8	14	8.6
Hellsten‐Westing et al. [116]	NC	11 (11/0)	53.0 ± −	23.6 ± 5.9	—	6	18	10	50	7.0	15	15	2.4
Allen et al. [44]	C	21 (9/12)	40.5 ± 4.0	49.2 ± 6.1	27.8 ± 4.5	9	27	10	180	6.5	10	24	13.4

*Note:* Studies are ordered by increasing number of sprint repetitions used in the intervention protocol.

Abbreviations: BM, body mass; BMI, body mass index; C, controlled; CC, crossover controlled; NC, not controlled.

**TABLE 2:** Summary statistics of moderators included in the meta‐regression.

Moderator	Mean ± SD	Min	Max
Age (years)	27.5 ± 8.8	20	64.3
Baseline V̇O_2_max (mL kg^−1^ min^−1^)	39.3 ± 7.1	21.6	53.5
BMI (kg m^−2^)	25.2 ± 2.4	21	35.7
Number of sprints	6 ± 3	2	24
Intervention duration (weeks)	5 ± 2	2	12
Weekly session frequency (sessions week^−1^)	3 ± 0.5	2	5
Work: rest ratio	0.134 ± 0.062	0.03	0.5
Total weekly sprint time (s week^−1^)	431 ± 226	60	960

**TABLE 3:** Estimated changes in V̇O_2_max over meaningful moderator changes.

Moderator	Contrast used	Estimated change in V̇O_2_max (%)	95% CrI (%)
Sprint repetitions	+2 sprint repetitions	−0.09	−0.64 to 0.48
Sprint duration	+10 s sprint duration	0.29	−0.92 to 1.55
Intervention duration	+4 weeks intervention duration	2.69	1.25 to 4.19
Training frequency	+1 session week^−1^	1.19	−0.41 to 2.77
Male participants	+10 percentage‐points male	−0.09	−0.38 to 0.20
Age	+10 years	−0.89	−1.94 to 0.16
Baseline VO_2_max	+3.5 mL kg^−1^ min^−1^ baseline VO_2_max (1 MET)	−0.32	−0.86 to 0.21
Work: rest ratio	+2 SD work: rest ratio (0.123)	0.03	−0.89 to 0.96
BMI	+2 SD BMI (4.9 kg m^−2^)	0.07	−2.07 to 2.14
Total weekly sprint time	+120 s week^−1^	−0.01	−0.49 to 0.48

*Note:* Values represent estimated percentage changes in V̇O_2_max associated with practically meaningful moderator contrasts. Work: rest ratio and BMI were interpreted over 2 SD due to the absence of clearly defined practical units. BMI estimates were derived from a sensitivity model due to missing BMI data (*n* = 73).

**FIGURE 4:**
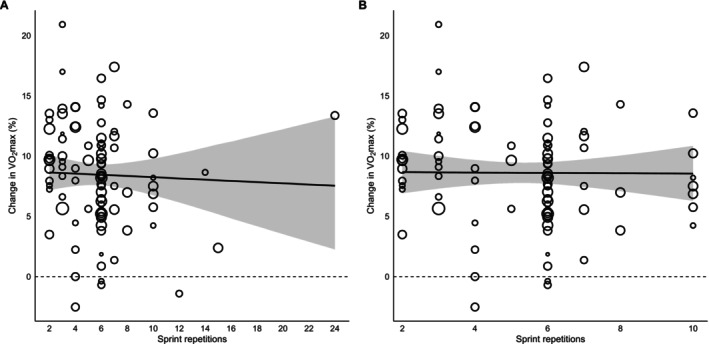
Association between the number of sprint repetitions per training session and changes in V̇O_2_max, including (A; *n* = 93) and excluding (B; *n* = 89) studies implementing > 10 sprints per session.

The risk of bias analysis indicated that there was a moderate risk of bias overall. This result was largely determined by “some concerns” in three of the seven domains (Selection of the reported result, Measurement of the outcome, and Randomization process; File S1 and Figure 4a–c) assessed across the three risk of bias tools. The issues within the three domains were due to lack of study preregistration, non‐blinding of the outcome assessors, and the absence of randomization in some studies. Individual study results from the three risk of bias analysis tools can be seen in File S1 and Figure 4d,e.

Figure 4a illustrates the association between the number of sprint repetitions and changes in V̇O_2_max. Estimates were centred near zero, indicating a trivial negative association (−0.04% per additional sprint; 95% CrI: −0.32% to 0.24%), with uncertainty spanning both small positive and negative values. For example, an increase of four sprints corresponds to an estimated change of approximately −0.2%, which is trivial in magnitude. Figure 4b displays the results of a sensitivity analysis in which atypical SIT protocols incorporating > 10 sprints per session were excluded (*n* = 4). Removing these studies resulted in a slightly larger pooled estimate (8.5%, 95% CrI: 7.2% to 9.7%) and a weaker association between sprint repetitions and changes in V̇O_2_max (−0.01% per sprint; 95% CrI: −0.43% to 0.41%). However, estimates remained centred around zero with substantial uncertainty, and the overall interpretation remained unchanged.

**FIGURE 5:**
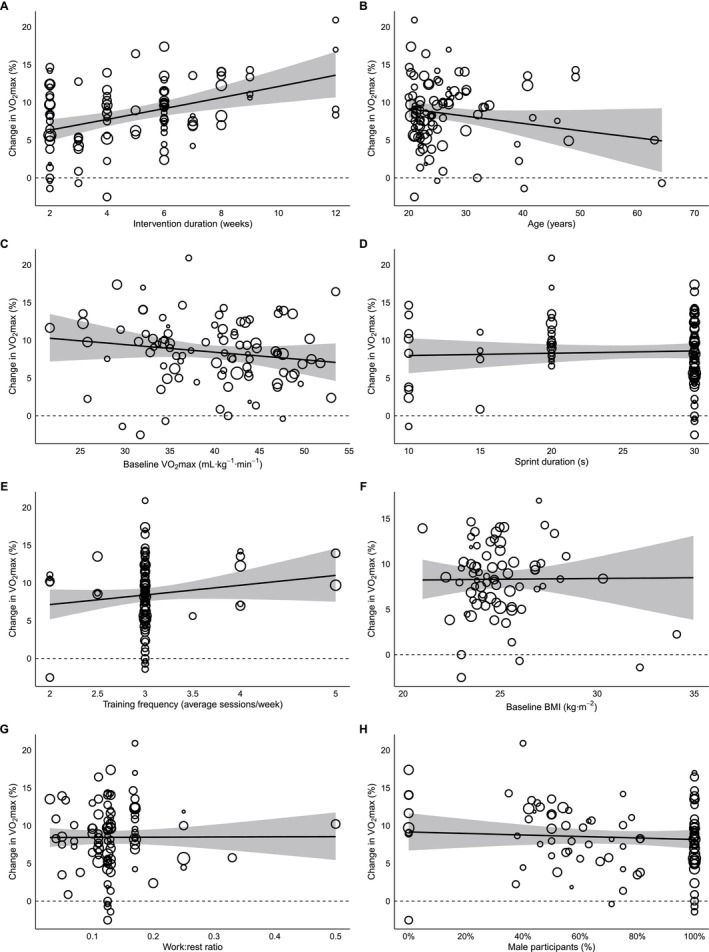
Associations between study‐level moderators and changes in V̇O_2_max: Intervention duration (A), age (B), baseline V̇O_2_max (C), sprint duration (D), training frequency (E), BMI (F), work: rest ratio (G), and percentage of male participants (H). BMI data were available for 73 trials due to missing data. Training frequency was modeled and displayed as a continuous moderator; values of 2.5 and 3.5 sessions week^−1^ represent studies reporting variable frequencies of 2–3 and 3–4 sessions week^−1^, respectively.

**FIGURE 6:**
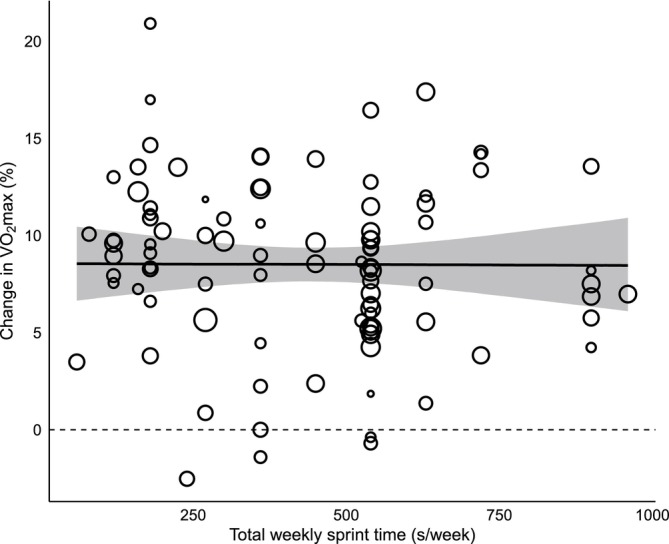
Association between total weekly sprint time and changes in V̇O_2_max.

Each additional week of SIT (Figure 5a) was associated with a small, positive change in V̇O_2_max (0.7% per week; 95% CrI: 0.3% to 1.0%). Conversely, age (Figure 5b) and baseline V̇O_2_max (Figure 5c) showed a trivial negative association (−0.09% per year; 95% CrI: −0.20% to 0.02% and −0.09% per mL kg^−1^ min^−1^; 95% CrI: −0.25% to 0.06%, respectively), although credible intervals spanned both negative and positive values, indicating no consistent directional association. For sprint duration (0.03% per second; 95% CrI: −0.09% to 0.15%; Figure 5d), training frequency (1.19% per session week^−1^; 95% CrI: −0.41% to 2.77%; Figure 5e), BMI (0.01 per kg m^−2^; 95% CrI: −0.43% to 0.44%; Figure 5f), work: rest ratio (0.24%; 95% CrI: −7.03% to 8.09%; Figure 5g), sex (−0.92%; 95% CrI: −3.74% to 2.02%; Figure 5h), and study design (0.40%; 95% CrI: −1.25% to 2.06%; Table S1) estimated associations were highly uncertain, with credible intervals spanning positive and negative values. As such, these variables do not show a consistent directional association with changes in V̇O_2_max. Figure 6 illustrates the association between total weekly sprint time and changes in V̇O_2_max (−0.0001%; 95% CrI: −0.004% to 0.004%). Estimated changes were trivial and uncertain, with credible intervals spanning both positive and negative values, indicating no consistent directional association.

## 5 Discussion

The main aim of the present study was to provide a robust estimate for the modifying effect of the number of sprint repetitions per training session in SIT protocols on the training‐induced increase in V̇O_2_max in untrained individuals. The present study extends the previous meta‐analysis [34] in two ways: (1) by employing a Bayesian hierarchical modeling framework, and (2) improving statistical power by more than doubling the number of trials included in the analysis. Magnitude‐Based Inference has been the subject of considerable methodological debate. Critics have raised concerns regarding its statistical properties, including the potential for inflated Type I error rates and the interpretation of uncertainty estimates [36, 37, 38]. Whereas others have argued that it represents a valid Bayesian approach for making inferences about interval hypotheses and practical significance [39]. The present study adopted a Bayesian hierarchical modeling framework that directly quantified uncertainty through posterior distributions and credible intervals, while allowing the inclusion of nested random effects and multiple moderators within a single model. Using data from 78 studies with 93 trials, involving a total of *n* = 1188 participants, we extend previous findings [34] by showing that increasing the number of sprint repetitions in a SIT protocol is not associated with meaningful additional improvements in V̇O_2_max. Consistent with Vollaard, Metcalfe and Williams [34], estimates in the present analysis also suggested a negative association between sprint repetitions and improvements in V̇O_2_max. However, the magnitude of this association was trivial and highly uncertain, with credible intervals spanning both negative and positive values, reinforcing the broader conclusion that increasing sprint repetitions does not meaningfully augment training adaptations. To aid interpretation of the meta‐regression findings, model estimates were translated into practically meaningful moderator changes. Increasing intervention duration by four weeks was associated with the largest estimated improvement in V̇O_2_max (2.7%), whereas increasing the number of sprints by two was associated with a trivial change (−0.09%). This supports the interpretation that extending the training intervention may be more influential than increasing the number of sprint repetitions within individual SIT sessions. Other moderators, including sprint duration, training frequency, baseline V̇O_2_max, sex distribution, work: rest ratio, total weekly sprint time, and BMI, showed small or uncertain associations when expressed over meaningful contrasts, with credible intervals spanning both positive and negative values. Taken together, these findings indicate that reducing the number of sprint repetitions does not meaningfully attenuate improvements in V̇O_2_max. This is clinically important given the substantial evidence showing that V̇O_2_max is among the most powerful independent predictors of all‐cause and cardiovascular mortality [117]. The practical implication of our finding is that reducing the number of sprint repetitions while maintaining worthwhile improvements in V̇O_2_max may have potential implications for the feasibility and acceptability of low‐volume SIT. For example, whereas the classic SIT protocol involving 4–6 × 30‐s sprints takes ~30 min to complete [92, 100, 104, 118], a REHIT session involving 2 × 20‐s sprint repetitions takes just 10 min [20, 21]. Additionally, performing fewer sprint repetitions has been shown to attenuate the drop in affective valence during SIT [35]. Taken together with the present finding that reducing sprint repetitions does not compromise improvements in V̇O_2_max, this suggests that low‐volume SIT may represent a viable approach to improving health. However, the present analysis did not directly assess perceptual or behavioral responses, and these implications should therefore be interpreted with caution. Since improvements in V̇O_2_max are most commonly achieved through regular structured exercise, the significance of developing alternative interventions that promote greater adoption to regular exercise remains an important priority.

The mechanisms underlying adaptations to SIT are not fully understood. The present analysis showed that the estimated effect of sprint repetitions was centred close to zero, indicating little change in V̇O_2_max per additional sprint. Importantly, this estimate reflects the average change per additional sprint around the mean number of sprints performed across studies, rather than being relative to a specific reference point (e.g., two sprints). As such, the present model does not allow direct inference regarding the minimum number of sprints required to elicit adaptations. Previous work has proposed that a substantial portion of the adaptive stimulus may be achieved with just two sprints supramaximal efforts. For example, rapid glycogen depletion during initial sprints has been suggested to trigger signaling pathways associated with aerobic adaptation [34], with evidence indicating that glycogenolysis is markedly reduced after two supramaximal sprints [119]. This early metabolic disturbance may also contribute to a disruption of circulatory homeostasis, providing a stimulus for increases in blood volume, which has been proposed as a key central adaptation underlying improvements in V̇O_2_max following SIT [34, 73]. It was also suggested that release and activation of glycogen‐bound 5′‐AMPK associated with glycogenolysis could trigger signaling pathways associated with peripheral adaptations such as mitochondrial biogenesis and enhanced oxidative capacity, contributing to aerobic adaptations [34]. Thus, performing more than 2 sprint repetitions may not further activate key signaling processes required for training adaptations, but will make the training session longer, more tiring, and more unpleasant. In line with this, the present findings indicate no clear dose–response relationship between the number of sprint repetitions per session and improvements in V̇O_2_max, suggesting that additional sprint repetitions may provide limited incremental benefit. However, a minimum effective does (e.g., two sprints) cannot be directly inferred from the current analysis and therefore remains to be determined. The minimum effective dose of SIT remains uncertain, to our knowledge, only one study has investigated the effect of a single all‐out sprint in a SIT intervention [120], reporting no significant change in V̇O_2_max in the training group. Combined with the absence of a clear dose–response relationship in the present analysis, this highlights the need for further research to define the lower boundary of the dose–response curve.

The present findings suggest that, of the exercise protocol parameters examined, intervention duration showed the clearest positive association with changes in V̇O_2_max following SIT. Each additional week of training was associated with an estimated increase of 0.7% in V̇O_2_max, and when expressed over a practically meaningful change (e.g., four weeks) this corresponds to an increase of ~2%–3%. However, it should be noted that the model assumes a linear relationship between intervention duration and changes in V̇O_2_max, whereas a plateau in adaptation is likely to occur over time. Although the majority of studies only measure V̇O_2_max pre‐ and post‐intervention, numerous studies have assessed the time‐course of changes in V̇O_2_max. Some of these have observed progressive increases in V̇O_2_max over time [27, 30, 94], but others [92, 97, 115] support comments by Sloth et al. [11] in their previous meta‐analysis, that some of the adaptations to SIT occur in the early stages of training and that the magnitude of improvements taper off. The reasons for the contrasting results are unclear. Conceptually, over short training periods there must be an effect of intervention duration while V̇O_2_max improves from baseline. However, in time, the rate of improvements will slow, and a “plateau” may be reached with no further improvements in V̇O_2_max [121], thus eliminating the ongoing positive effect of intervention duration. To date, it is unknown if, and when such a plateau will occur with different SIT protocols. As the longest intervention duration we identified in the current study is 12 weeks [27, 30, 31], further research exploring longer duration SIT interventions may help to determine the, presumably curvilinear, association between intervention duration and the magnitude of V̇O_2_max response.

One limitation to the findings of the present study relates to the potential for small‐study effects. While visual inspection of the funnel plot did not suggest meaningful asymmetry, and this was supported by a non‐significant Egger's regression test, it is acknowledged that funnel plot‐based methods have limitations and cannot definitively exclude bias [122, 123]. Sterne et al. [123] suggest the use of the term “small‐study effects” as an alternative way of describing the observed trend as there are a range of influences that may contribute to these small‐study effects such as outcome reporting bias, heterogeneity between studies, or other sources beyond publication bias alone [122]. There are limitations in relation to the review processes used in the present study as only PubMed and Web of Science were searched, while gray literature was not included, which may have contributed to the risk of publication bias. Missing V̇O_2_max data was extracted using digitisation software, which may have introduced error. Furthermore, sampling variance calculations required imputation of a pre‐post correlation coefficient (*r* = 0.90), although sensitivity analyses with different values suggested the findings were robust to this assumption. Additionally, the inclusion of non‐controlled studies may have contributed to increased heterogeneity and potentially produced wider uncertainty in model estimates compared with analyses limited to randomized‐controlled trials. An additional limitation is that studies implementing sprint durations < 10 s were excluded. This decision was made to maintain a relatively homogeneous definition of SIT centred on repeated all‐out Wingate‐type efforts and to minimize overlap with other forms of interval training that often employ very short sprint durations, such as RST, Tabata‐style exercise or approaches such as the 10‐20‐30 method. However, this exclusion criterion may have influenced the generalisability of the findings, as protocols using sprint durations < 10 s may differ in their physiological demands, recovery structure, and intended training outcomes, and may therefore elicit somewhat different physiological adaptations. Consequently, the present findings should be interpreted as applying specifically to SIT protocols involving all‐out sprints of ≥ 10 s duration and should not be extrapolated to other forms of sprint‐based interval training. Moreover, the present study focused solely on V̇O_2_max as an outcome measure. Therefore, the finding that increasing sprint repetitions shows no consistent association with changes in V̇O_2_max cannot be generalized across other important health markers such as blood pressure, body composition, blood lipid profiles or insulin sensitivity. There is a need for studies involving larger sample sizes, but we restate our previous recommendation [33] to focus on SIT protocols involving fewer/shorter sprints in these studies. Even if publication bias/small‐study effects may have masked a small but significant positive effect of the number of sprint repetitions on improvements on V̇O_2_max, the combination of effectiveness at improving key health markers [20, 22, 27, 28, 124] and measures of acceptability [19, 35, 125] suggests that low‐volume SIT protocols like REHIT may represent more practical candidates for real‐world implementation compared to classic SIT protocols.

In conclusion, using a more robust statistical approach and a larger dataset, we extend previous findings by corroborating the negative direction of the association between sprint repetitions and improvements in V̇O_2_max reported by Vollaard, Metcalfe, and Williams [34]. However, the estimated magnitude of this association was trivial and highly uncertain, providing little evidence that performing additional sprint repetitions meaningfully enhances training‐induced improvements in V̇O_2_max. These findings support the use of lower‐volume SIT protocols as an efficient approach to improving cardiorespiratory fitness and highlight the need for future research to identify the minimum effective dose for maximizing health benefits. The present findings suggest that low‐volume SIT protocols should be prioritized in future research examining their real‐world implementation as interventions for improving general health in inactive populations.

## 6 Perspective

The present systematic review and meta‐analysis provides timely and clinically relevant evidence for informing exercise medicine and public health practice. While prior syntheses have established SIT as an effective exercise intervention to improve V̇O_2_max, concerns have persisted regarding tolerability and time‐efficiency, along with the absence of clear physiological justification for the most commonly used “classic” SIT protocol (4–6 × 30 s sprints). The present findings demonstrate that increasing the number of sprint repetitions does not meaningfully augment improvements in V̇O_2_max in physically inactive adults. This finding extends previous work [34] by providing a more statistically robust and a stronger powered confirmation that low‐volume SIT protocols (2–3 × 20 s sprints) achieve comparable aerobic adaptations. Given the strong association between V̇O_2_max and all‐cause and cardiovascular mortality, even modest improvements are clinically important. The implication for sports medicine is clear: reducing sprint volume enhances time‐efficiency and likely improves acceptability without compromising physiological benefit. Therefore, future intervention studies and real‐world implementation strategies should prioritize low‐volume SIT as a pragmatic, scalable approach to improving cardiorespiratory fitness in untrained populations.

## Author Contributions

M.H., N.B.J.V., and R.S.M. conceived and designed the review. M.H. and N.B.J.V. conducted the literature search, screening, data extraction, and risk of bias screening. M.H., R.D.P., and I.J.G. performed and/or assisted in the statistical analyses. All authors contributed to the interpretation of the findings and revision of the manuscript. N.B.J.V. is the guarantor and accepts the full responsibility for the work and the conduct of the study.

## Funding

The authors have nothing to report.

## Ethics Statement

The authors have nothing to report.

## Consent

The authors have nothing to report.

## Conflicts of Interest

The authors declare no conflicts of interest.

## Supporting information


**Figure S1:** Trace plots for original model assessing convergence of model. Each panel represents a different regression coefficient sampled in the model.
**Table S1:** Posterior estimates from the main Bayesian hierarchical meta‐regression model.
**Table S2:** Prior sensitivity analysis model results.
**Figure S2:** (A) Posterior distribution of the pooled effect size. Black circle indicates posterior mean while black solid lines represent the 95% CrI. (B) Posterior distribution of between‐study heterogeneity. Black circle indicates posterior mean while solid black lines represent the 95% CrI. (C) Posterior Predictive Check plot.
**Figure S3:** (A) Posterior density plot for original and alternative prior models. (B) Posterior distribution of between‐study heterogeneity for original and alternative prior models.
**Table S3:** Sensitivity analysis model results excluding 4 outlier SIT studies.
**Figure S4a:** Risk of Bias assessment summary. RoB2.0 tool for randomized trials (v22.8.2019).
**Figure S4b:** Risk of Bias assessment summary. ROBINS‐I tool for non‐randomized trials (ROBINS‐I V2, 2024).
**Figure S4c:** Risk of Bias assessment summary. RoB 2 tool for crossover trials (RoB 2, v18.3.2021).
**Figure S4d:** Risk of Bias domain‐level risk assessment summary (RoB2.0 v22.8.2019).
**Figure S4e:** Domain‐level risk of bias assessment. ROBINS‐I for non‐randomized studies (ROBINS‐I V2, 2024).

## Data Availability

All relevant data is available within the article and its Supporting Information. Raw data file(s) and code used within the statistical analyses are available at: https://github.com/matthutch95/SIT_VO2max_Reproducibility.git.
