# Supplementary material for: Number of Sprint Repetitions in a Sprint Interval Training Session Does Not Moderate Improvements in V̇O2max With Training: A Systematic Review and Meta‐Analysis
Source: Scand J Med Sci Sports. 2026 Jul 20;36(7):e70347. doi: 10.1111/sms.70347 (PMC13385658; doi:10.1111/sms.70347)
Supplement: Supplementary file 1 — Figure S1: Trace plots for original model assessing convergence of model. Each panel represents a different regression coefficient sampled in the model. Table S1: Posterior estimates from the main Bayesian hierarchical meta‐regression model. Table S2: Prior sensitivity analysis model results. Figure S2: (A) Posterior distribution of the pooled effect size. Black circle indicates posterior mean while black solid lines represent the 95% CrI. (B) Posterior distribution of between‐study heterogeneity. Black circle indicates posterior mean while solid black lines represent the 95% CrI. (C) Posterior Predictive Check plot. Figure S3: (A) Posterior density plot for original and alternative prior models. (B) Posterior distribution of between‐study heterogeneity for original and alternative prior models. Table S3: Sensitivity analysis model results excluding 4 outlier SIT studies. Figure S4a: Risk of Bias assessment summary. RoB2.0 tool for randomized trials (v22.8.2019). Figure S4b: Risk of Bias assessment summary. ROBINS‐I tool for non‐randomized trials (ROBINS‐I V2, 2024). Figure S4c: Risk of Bias assessment summary. RoB 2 tool for crossover trials (RoB 2, v18.3.2021). Figure S4d: Risk of Bias domain‐level risk assessment summary (RoB2.0 v22.8.2019). Figure S4e: Domain‐level risk of bias assessment. ROBINS‐I for non‐randomized studies (ROBINS‐I V2, 2024). [file SMS-36-e70347-s001.docx]

**Supplementary Data**

**Number of sprint repetitions in a sprint interval training session does not moderate improvements in V̇O_2_max with training: a systematic review and meta-analysis**

Hutchinson M^1^, Kinghorn D^1^, Hall ECR^1^, Metcalfe RS^2^, Paval DR^1^, Gallagher IJ^3^, Vollaard NBJ^1*^

*^1^: Faculty of Health Sciences and Sport, University of Stirling, Stirling, UK*

*^2^: Applied Sports, Technology, Exercise and Medicine Research Centre (A-STEM), Swansea University, Swansea, UK*

*^3^: Centre for Biomedicine & Global Health, School of Applied Sciences, Edinburgh Napier University, Edinburgh, UK*

*: Corresponding author

Dr Niels BJ Vollaard

Faculty of Health Sciences and Sport

University of Stirling

Stirling, UK

[n.vollaard@stir.ac.uk](mailto:n.vollaard@stir.ac.uk)

*Search Strategy****PubMed Search Strategy:***

(“Wingate” OR “all-out” OR “sprint” OR “interval training”)

AND
(“VO2max” OR “VO2peak” OR “aerobic capacity” OR “oxygen uptake” OR “aerobic power”)

***Web of Science Search Strategy****:*

(“Wingate” OR “all-out” OR “sprint” OR “interval training”)

AND
(“VO2max” OR “VO2peak” OR “aerobic capacity” OR “oxygen uptake” OR “aerobic power”)

Date range: 01/05/2016 to 30/09/2024

*Bayesian Model Specifications*

Harrer et al [1] describe the assumptions of the model. Weakly informative priors were used as recommended by the developers of the Stan probabilistic language upon which *brms* is based [2]. A normally distributed prior (0, 0.15) was specified for the intercept, and for all moderator coefficients (0, 0.05). Between-study heterogeneity (τ) was modelled using an exponential prior (exponential(2)) applied to the SD of the random effect. These priors weakly informative and selected to aid model estimation and avoid implausible values, allowing flexibility for the data to dominate the posterior distributions. Given the relatively large number of included studies, posterior estimates were expected to be largely data-driven, with priors having minimal influence beyond improving computational stability. Hamiltonian Markov Chain Monte Carlo (MCMC) methods [3] within the statistical package *brms* [4] were used to estimate the posterior distribution of the overall effect and for each parameter. Four chains, each with 5,000 iterations were run for the sampling, 500 iterations were designated as warm-up iterations, allowing the chains to stabilise before sampling [5], resulting in a total of 18,000 posterior samples across all chains.

Study parameters and participant characteristics were added as moderators to the model to assess the influence they each had on the overall pooled effect size. The following moderators were included: number of sprint repetitions, sprint duration (s), intervention duration (weeks), work:rest ratio, baseline relative V̇O_2_max (mL·kg^-1^·min^-1^), age (y), training frequency (sessions/week), sex (% male), study design (controlled/uncontrolled), total weekly sprint time (s/week), and BMI (kg·m^-2^). All reported model estimates are posterior means and 95% credible intervals (CrIs) where the true effect is estimated to lie with 95% probability [6]. Conditional effects plots were used to visualise the relationships between effect size and the moderators.

*Model Checks*

A posterior predictive check (PPC) was conducted to assess the fit of the model; a PPC plot was created to visualise whether the simulated data run by the model closely resembles the observed data [7, 8]. Between-study heterogeneity (τ) was assessed within the model and summarised using a posterior density plot with 95% CrIs. Convergence was assessed by visually assessing trace plots (**Supplementary Figure 1**) and confirming that ‘*rhat*’ [9] values were all close to 1 [10].

*Sensitivity Analyses*

A prior sensitivity analysis was performed to demonstrate that posterior estimates were not meaningfully influenced by prior specification. [11, 12]. A more diffuse prior was applied (normal (0, 0.30); normal (0, 0.10); heterogeneity (exponential(1)), and resulting posterior estimates were compared with those from the primary model. This choice was based on general guidelines provided that suggest if diffuse priors were used in the original analysis, then different forms of diffuse priors would be recommended for the sensitivity analysis [13]. A sensitivity analysis was carried out by removing 4 outlier datasets that contained >10 sprint repetitions, as all these datasets involved short (10-15 s) sprints.

Supplementary Results

**Supplementary** **Figure 2a** illustrates the results for the posterior probability distribution of the pooled effect size. There was a small amount of heterogeneity between studies (τ*_between_* = 0.018; 95% CI = 0.002 - 0.031; **Supplementary Figure 2b**). **Supplementary Figure 2c** demonstrates that the model is a good fit for the given data as there is good alignment between observed and simulated data. Results of the prior sensitivity analysis model (**Supplementary Table 2)** were similar to the original model (**Supplementary Table 1**). **Supplementary Figure 3** illustrates the similarities between the primary model and the prior sensitivity analysis model for both the posterior distribution of the pooled effect size (**Supplementary Figure 3a**) and between-study heterogeneity (**Supplementary Figure 3b)**. The near-complete overlap between posterior distributions indicates that estimates were largely unaffected by prior specification and were primarily being driven by the observed data [13]. This is consistent with expectations given the number of included studies [14]. Moderator effect estimates (**Supplementary Tables 1-2**) were similar across models, indicating that inferences were consistent across prior specifications.

**Supplementary Table 3** shows


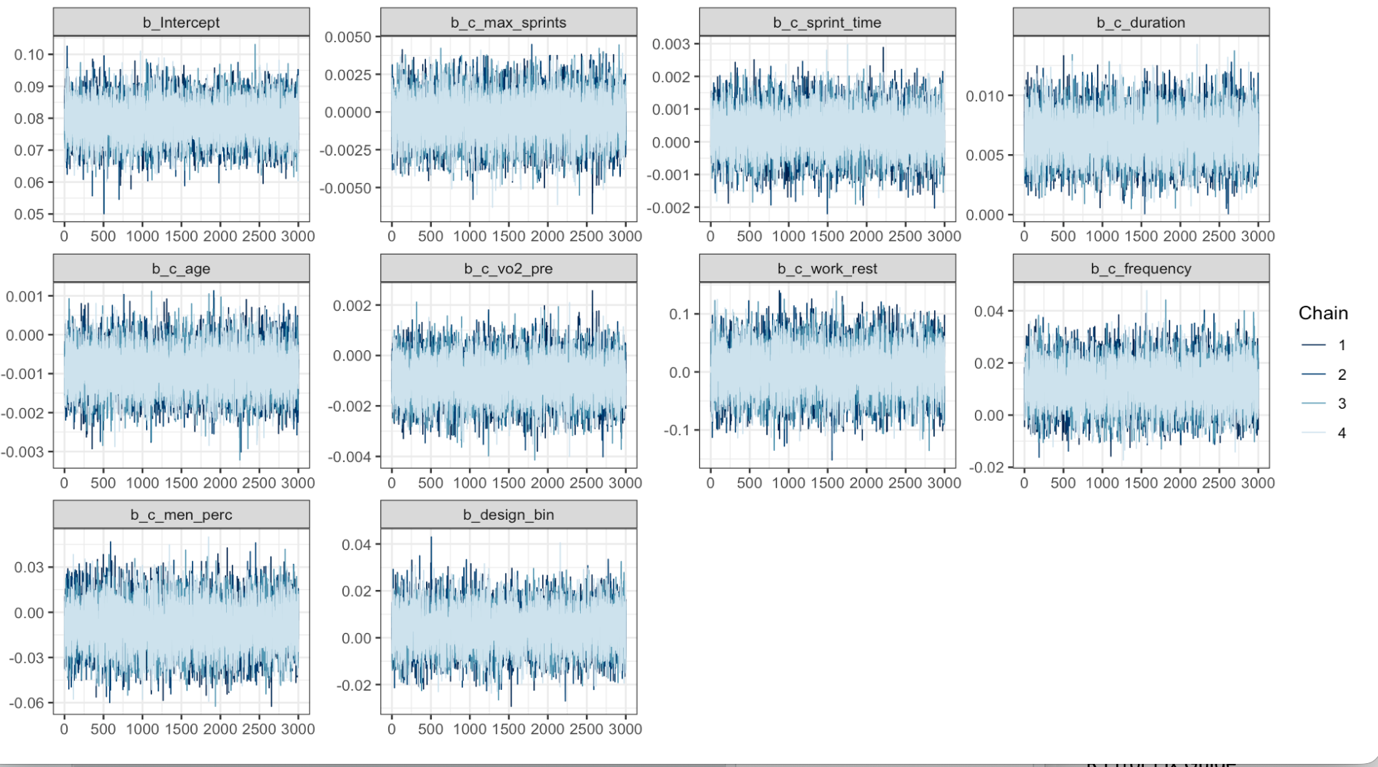


**Supplementary Fig. 1** – *Trace plots for original model assessing convergence of model. Each panel represents a different regression coefficient sampled in the model*

**Supplementary Table 1** – Posterior estimates from the main Bayesian hierarchical meta-regression model.

| **Parameter** | **Estimate** | **Est. Error** | **l-95% CrI** | **u-95% CrI** | **Estimate %** | **l-95% CrI (%)** | **u-95% CrI (%)** | **Rhat** | **Bulk_ESS** | **Tail_ESS** |
| --- | --- | --- | --- | --- | --- | --- | --- | --- | --- | --- |
| Intercept | 0.0793 | 0.0058 | 0.0679 | 0.0906 | 8.25 | 7.03 | 9.48 | 1 | 14249 | 9635 |
| Sprint Repetitions | -0.0004 | 0.0014 | -0.0032 | 0.0024 | -0.04 | -0.32 | 0.24 | 1 | 14500 | 10366 |
| Sprint duration (s) | 0.0003 | 0.0006 | -0.0009 | 0.0015 | 0.03 | -0.09 | 0.15 | 1 | 13759 | 10500 |
| Intervention duration (weeks) | 0.0066 | 0.0018 | 0.0031 | 0.0103 | 0.67 | 0.31 | 1.03 | 1 | 13091 | 10098 |
| Age (y) | -0.0009 | 0.0005 | -0.0020 | 0.0002 | -0.09 | -0.20 | 0.02 | 1 | 13805 | 11119 |
| Baseline V̇O_2_max (mL·kg^-1^·min^-1^) | -0.0009 | 0.0008 | -0.0025 | 0.0006 | -0.09 | -0.25 | 0.06 | 1 | 13914 | 10398 |
| Work:rest ratio | 0.0024 | 0.0386 | -0.0729 | 0.0778 | 0.24 | -7.03 | 8.09 | 1 | 16282 | 8835 |
| Training frequency | 0.0118 | 0.0080 | -0.0041 | 0.0273 | 1.19 | -0.41 | 2.77 | 1 | 14770 | 9637 |
| Gender (% male) | -0.0092 | 0.0148 | -0.0381 | 0.0200 | -0.92 | -3.74 | 2.02 | 1 | 13969 | 9449 |
| Study design | 0.0040 | 0.0084 | -0.0126 | 0.0204 | 0.40 | -1.25 | 2.06 | 1 | 13539 | 9584 |

Estimates are presented as log-transformed proportional changes in V̇O_2_max. For interpretability, estimates are also expressed as percentage changes. 95% CrI represent 95% uncertainty intervals. Rhat values indicate model convergence, and Bulk and Tail effective sample sizes (ESS) reflect sampling efficiency. All continuous moderators were mean-centred.

**Supplementary Table 2** – *Prior sensitivity analysis model results.*

| **Parameter** | **Estimate** | **Est. Error** | **l-95% CrI** | **u-95% CrI** | **Estimate %** | **l-95% CrI (%)** | **u-95% CrI (%)** | **Rhat** | **Bulk_ESS** | **Tail_ESS** |
| --- | --- | --- | --- | --- | --- | --- | --- | --- | --- | --- |
| Intercept | 0.0792 | 0.0057 | 0.0678 | 0.0906 | 8.24 | 7.02 | 9.48 | 1 | 12809 | 9763 |
| Sprint Repetitions | -0.0004 | 0.0014 | -0.0032 | 0.0023 | -0.04 | -0.32 | 0.24 | 1 | 14488 | 10801 |
| Sprint duration (s) | 0.0003 | 0.0006 | -0.0010 | 0.0015 | 0.03 | -0.10 | 0.15 | 1 | 14093 | 10930 |
| Intervention duration (weeks) | 0.0066 | 0.0018 | 0.0031 | 0.0103 | 0.66 | 0.31 | 1.04 | 1 | 11648 | 9863 |
| Age (y) | -0.0009 | 0.0005 | -0.0020 | 0.0002 | -0.09 | -0.19 | 0.02 | 1 | 13708 | 10609 |
| Baseline V̇O_2_max (mL·kg^-1^·min^-1^) | -0.0009 | 0.0008 | -0.0025 | 0.0007 | -0.09 | -0.25 | 0.07 | 1 | 13012 | 10309 |
| Work:rest ratio | 0.0042 | 0.0539 | -0.1019 | 0.1092 | 0.42 | -9.69 | 11.54 | 1 | 144458 | 9717 |
| Training frequency | 0.0123 | 0.0083 | -0.0040 | 0.0288 | 1.24 | -0.40 | 2.92 | 1 | 14976 | 9731 |
| Gender (% male) | -0.0097 | 0.0152 | -0.0393 | 0.0203 | -0.97 | -3.85 | 2.05 | 1 | 12590 | 9549 |
| Study design | 0.0041 | 0.0084 | -0.0124 | 0.0206 | 0.41 | -1.23 | 2.08 | 1 | 11399 | 9562 |

**Supplementary Fig. 2** - (**A**) Posterior distribution of the pooled effect size. Black circle indicates posterior mean while black solid lines represent the 95% CrI. (**B**) Posterior distribution of between-study heterogeneity. Black circle indicates posterior mean while solid black lines represent the 95% CrI. (**C**) Posterior Predictive Check plot


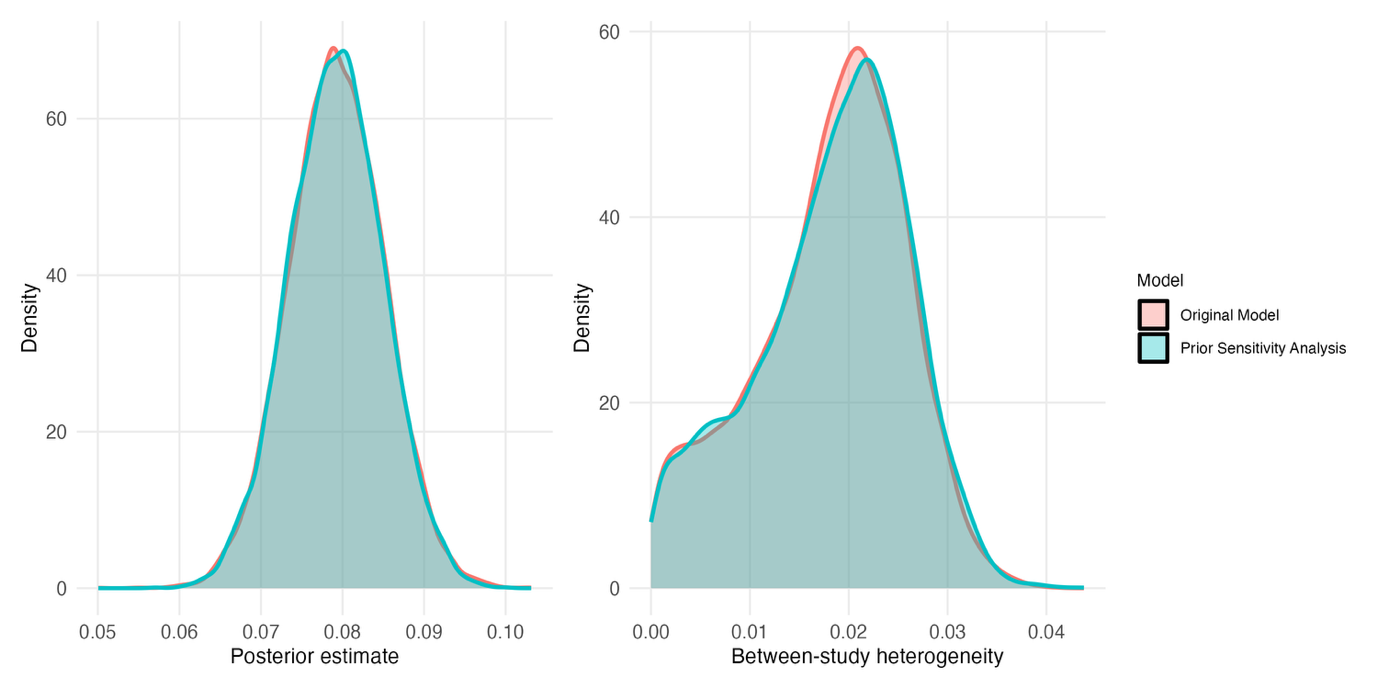


**Supplementary Fig. 3** – (**A**) *Posterior density plot for original and alternative prior models. (****B****) Posterior distribution of between-study heterogeneity for original and alternative prior models.*

**Supplementary Table 3** – *Sensitivity analysis model results excluding 4 outlier SIT studies.*

| **Parameter** | **Estimate** | **Est. Error** | **l-95% CrI** | **u-95% CrI** | **Estimate %** | **l-95% CrI (%)** | **u-95% CrI (%)** | **Rhat** | **Bulk_ESS** | **Tail_ESS** |
| --- | --- | --- | --- | --- | --- | --- | --- | --- | --- | --- |
| Intercept | 0.0811 | 0.0058 | 0.0698 | 0.0923 | 8.45 | 7.23 | 9.67 | 1 | 12910 | 9799 |
| Sprint Repetitions | -0.0001 | 0.0021 | -0.0043 | 0.0041 | -0.01 | -0.43 | 0.41 | 1 | 12435 | 10298 |
| Sprint duration (s) | -0.0001 | 0.0007 | -0.0015 | 0.0014 | -0.01 | -0.15 | 0.14 | 1 | 11301 | 10796 |
| Intervention duration (weeks) | 0.0060 | 0.0018 | 0.0025 | 0.0097 | 0.61 | 0.25 | 0.97 | 1 | 10910 | 9146 |
| Age (y) | -0.0009 | 0.0005 | -0.0020 | 0.0002 | -0.09 | -0.20 | 0.02 | 1 | 12686 | 11016 |
| Baseline V̇O_2_max (mL·kg^-1^·min^-1^) | -0.0009 | 0.0008 | -0.0024 | 0.0007 | -0.09 | -0.24 | 0.07 | 1 | 11500 | 9408 |
| Work:rest ratio | 0.0088 | 0.0392 | -0.0675 | 0.0858 | 0.88 | -6.53 | 8.96 | 1 | 15984 | 9298 |
| Training frequency | 0.0129 | 0.0083 | -0.0032 | 0.0294 | 1.30 | -0.32 | 2.99 | 1 | 12548 | 9029 |
| Gender (% male) | -0.0074 | 0.0149 | -0.0365 | 0.0225 | -0.74 | -3.58 | 2.27 | 1 | 11298 | 8952 |
| Study design | 0.0028 | 0.0084 | -0.0135 | 0.0191 | 0.28 | -1.34 | 1.93 | 1 | 11586 | 9961 |

**Supplementary Fig. 4a** – Risk of Bias assessment summary. RoB2.0 tool for randomised trials *(v22.8.2019)*


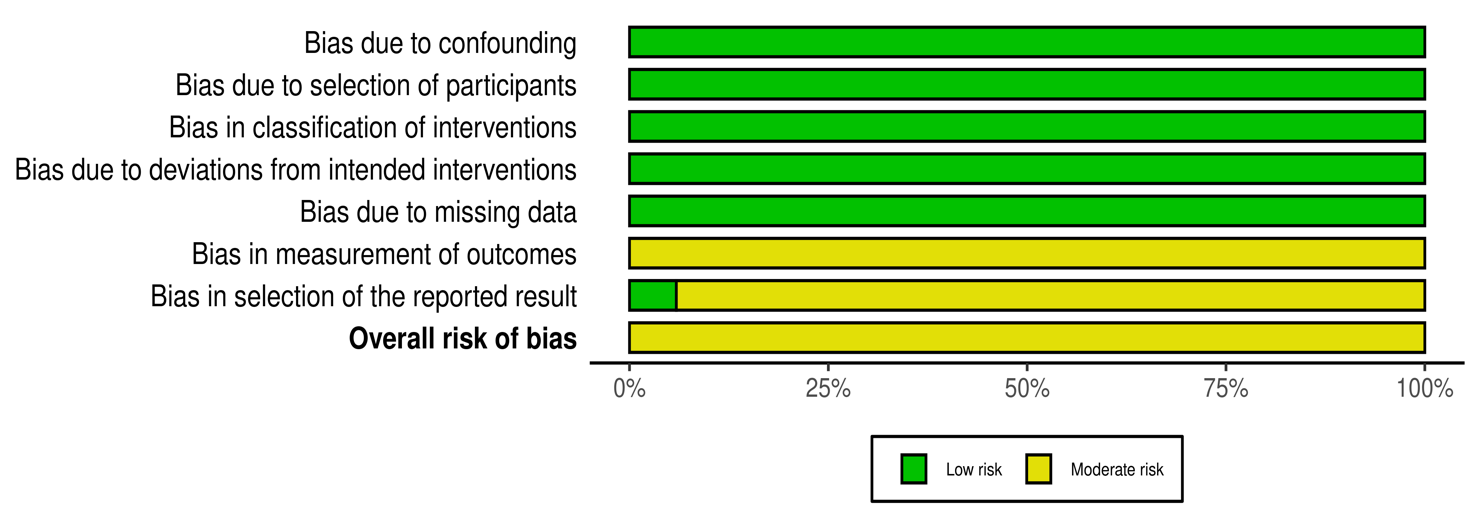


**Supplementary Fig. 4b** – Risk of Bias assessment summary. ROBINS-I tool for non-randomised trials (*ROBINS-I V2, 2024*)

**Supplementary Fig. 4c** – Risk of Bias assessment summary. RoB 2 tool for crossover trials. (*RoB 2, v18.3.2021*


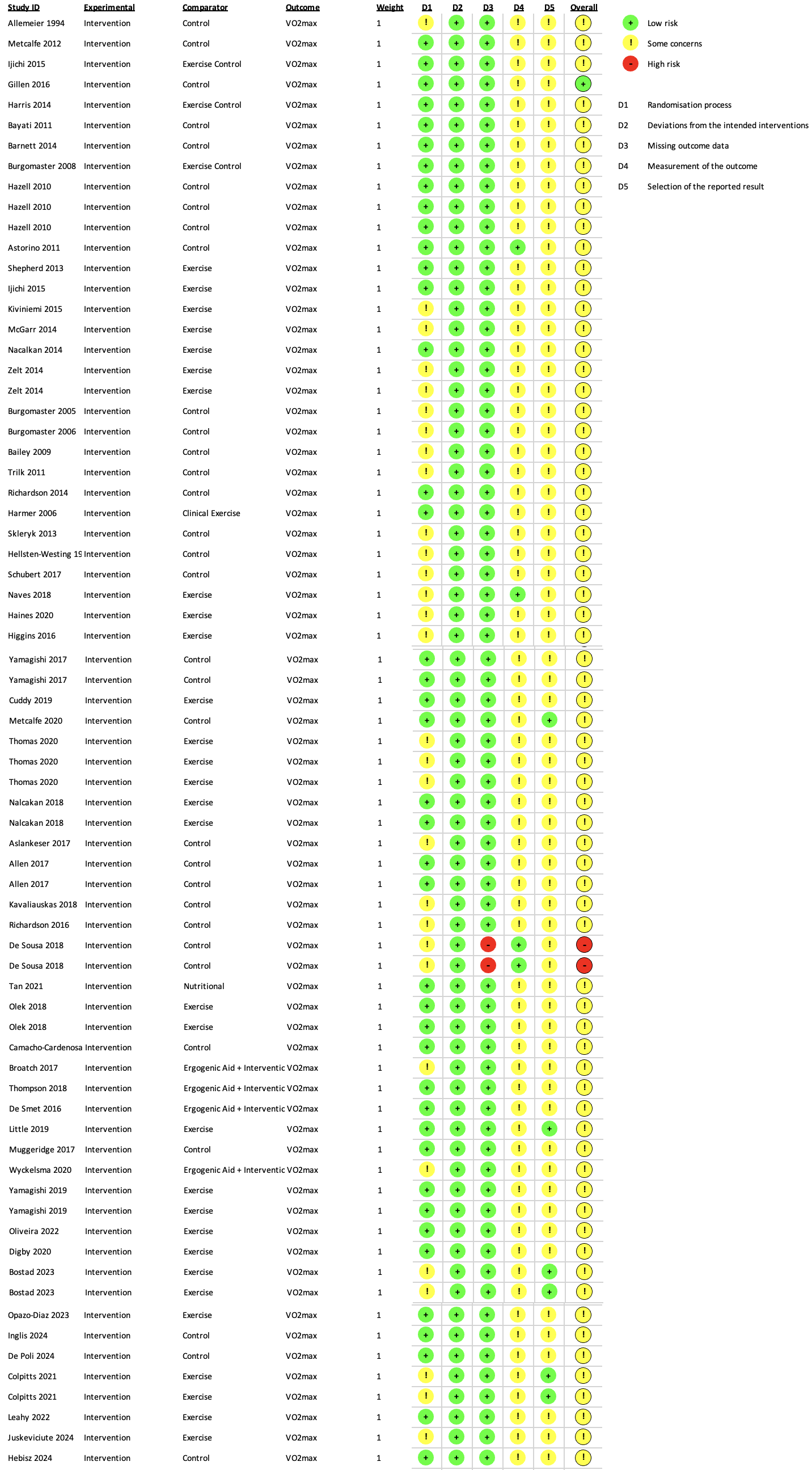


**Supplementary Fig. 4d** – Risk of Bias domain-level risk assessment summary. (*RoB2.0 v22.8.2019*)

*
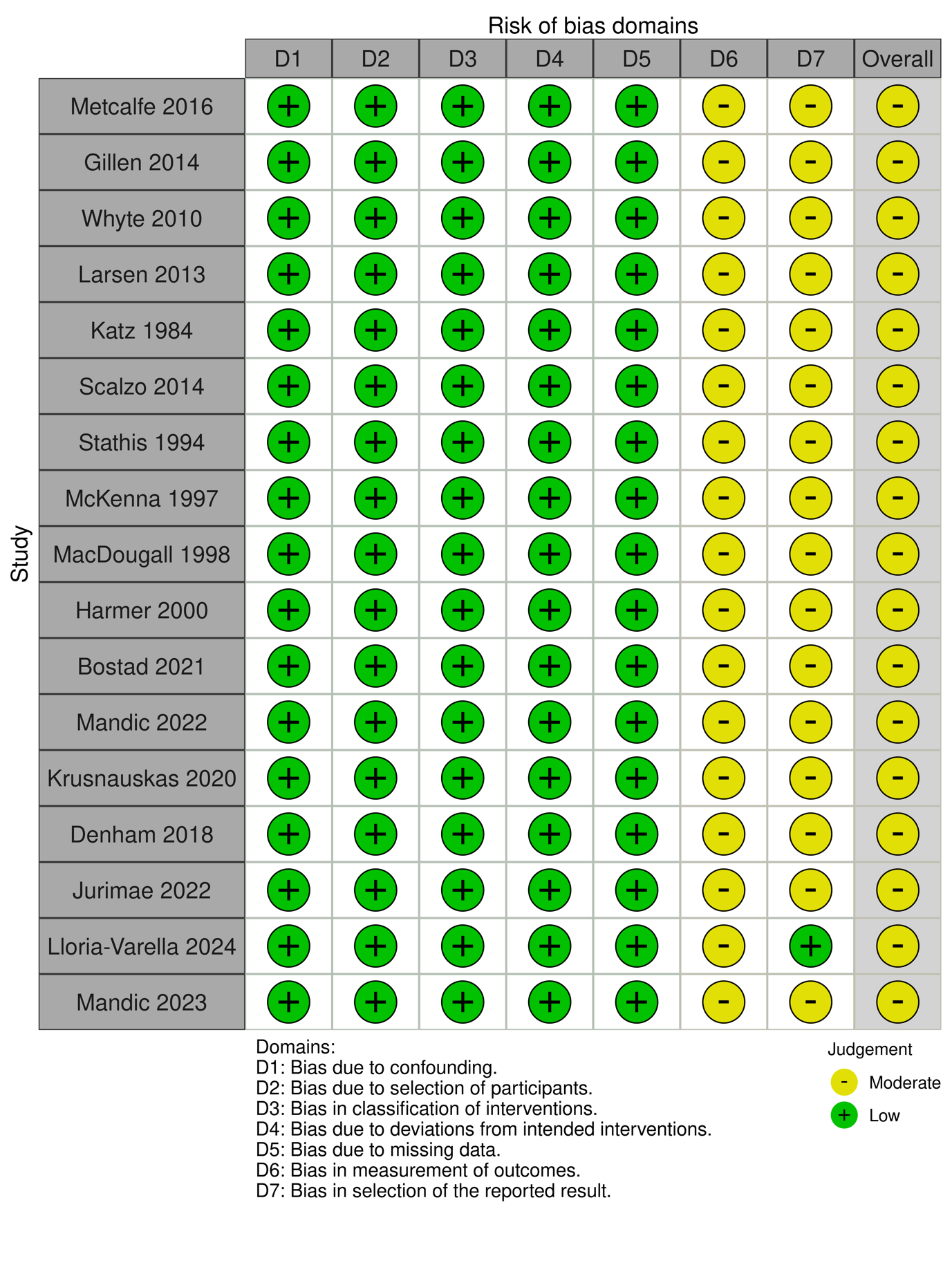
*

**Supplementary Fig. 4e** – Domain-level risk of bias assessment. ROBINS-I for non-randomised studies (*ROBINS-I V2, 2024*).

**References**

1. Harrer M, Cuijpers P, Furukawa T, et al. Doing Meta-Analysis with R. 1st ed. New York: Chapman and Hall/CRC; 2021 14 September 2021. 500 p.

2. Stan Development Team. Prior choice recommendations: Stan Project Wiki; 2025 [cited 2025 May 27]. Available from: <https://github.com/stan-dev/stan/wiki/Prior-Choice-Recommendations>.

3. Carpenter B, Gelman A, Hoffman MD, et al. Stan: A Probabilistic Programming Language. J Stat Softw. 2017;76.

4. Burkner P-C. brms : An R Package for Bayesian Multilevel Models Using Stan. Journal of Statistical Software. 2017;80(1):1-28.

5. Rindskopf D. Reporting Bayesian Results. Eval Rev. 2020;44(4):354-75.

6. Hespanhol L, Vallio CS, Costa LM, et al. Understanding and interpreting confidence and credible intervals around effect estimates. Braz J Phys Ther. 2019;23(4):290-301.

7. Kruschke JK. Posterior predictive checks can and should be Bayesian: comment on Gelman and Shalizi, 'Philosophy and the practice of Bayesian statistics'. Br J Math Stat Psychol. 2013;66(1):45-56.

8. Gabry J, Simpson D, Vehtari A, et al. Visualization in Bayesian Workflow. Journal of the Royal Statistical Society Series A: Statistics in Society. 2019;182(2):389-402.

9. Gelman A, Rubin DB. Inference from Iterative Simulation Using Multiple Sequences. Statistical Science. 1992;7(4):457-72, 16.

10. Vehtari A, Gelman A, Simpson D, et al. Rank-Normalization, Folding, and Localization: An Improved Rˆ for Assessing Convergence of MCMC (with Discussion). Bayesian Analysis. 2019;16(2):667-718.

11. van de Schoot R, Sijbrandij M, Depaoli S, et al. Bayesian PTSD-Trajectory Analysis with Informed Priors Based on a Systematic Literature Search and Expert Elicitation. Multivariate Behav Res. 2018;53(2):267-91.

12. Kallioinen N, Paananen T, Bürkner P-C, et al. Detecting and diagnosing prior and likelihood sensitivity with power-scaling. Statistics and Computing. 2023;34(1):57.

13. Depaoli S, Winter SD, Visser M. The Importance of Prior Sensitivity Analysis in Bayesian Statistics: Demonstrations Using an Interactive Shiny App. Front Psychol. 2020;11:608045.

14. Lambert PC, Sutton AJ, Burton PR, et al. How vague is vague? A simulation study of the impact of the use of vague prior distributions in MCMC using WinBUGS. Stat Med. 2005;24(15):2401-28.
